# Standardized description of scientific evidence using the Evidence Ontology (ECO)

**DOI:** 10.1093/database/bau075

**Published:** 2014-07-21

**Authors:** Marcus C. Chibucos, Christopher J. Mungall, Rama Balakrishnan, Karen R. Christie, Rachael P. Huntley, Owen White, Judith A. Blake, Suzanna E. Lewis, Michelle Giglio

**Affiliations:** ^1^Institute for Genome Sciences, University of Maryland School of Medicine, Baltimore, MD 21201, USA, ^2^Department of Microbiology and Immunology, University of Maryland School of Medicine, Baltimore, MD 21201, USA, ^3^Genomics Division, Lawrence Berkeley National Laboratory, Berkeley, CA 94720, USA, ^4^*Saccharomyces* Genome Database, Department of Genetics, Stanford University, Stanford, CA 94305, USA, ^5^Computational Biology and Bioinformatics, The Jackson Laboratory, Bar Harbor, ME 04609, USA, ^6^European Molecular Biology Laboratory, European Bioinformatics Institute (EMBL-EBI), Wellcome Trust Genome Campus, Hinxton, Cambridge CB10 1SD UK, ^7^Department of Epidemiology, University of Maryland School of Medicine, Baltimore, MD 21201, USA and ^8^Department of Medicine, University of Maryland School of Medicine, Baltimore, MD 21201, USA

## Abstract

The Evidence Ontology (ECO) is a structured, controlled vocabulary for capturing evidence in biological research. ECO includes diverse terms for categorizing evidence that supports annotation assertions including experimental types, computational methods, author statements and curator inferences. Using ECO, annotation assertions can be distinguished according to the evidence they are based on such as those made by curators versus those automatically computed or those made via high-throughput data review versus single test experiments. Originally created for capturing evidence associated with Gene Ontology annotations, ECO is now used in other capacities by many additional annotation resources including UniProt, Mouse Genome Informatics, *Saccharomyces* Genome Database, PomBase, the Protein Information Resource and others. Information on the development and use of ECO can be found at http://evidenceontology.org. The ontology is freely available under Creative Commons license (CC BY-SA 3.0), and can be downloaded in both Open Biological Ontologies and Web Ontology Language formats at http://code.google.com/p/evidenceontology. Also at this site is a tracker for user submission of term requests and questions. ECO remains under active development in response to user-requested terms and in collaborations with other ontologies and database resources.

**Database URL:** Evidence Ontology Web site: http://evidenceontology.org

## Introduction

An essential component of scientific research is the documentation of evidence-based conclusions resulting from investigations. Just as careful documentation of scientific methodology can allow other investigators to understand or reproduce an experiment, describing the scientific evidence associated with a given assertion or inference allows for its meaningful assessment. Many of the data that are used to support assertions are experimentally derived in a laboratory or field setting. However, assertions are also commonly made based on computational analysis of large data sets, implied by known biology and captured during literature curation, or even synthesized from investigator speculation. Because types of evidence can vary, it is essential to document this information to allow others to draw meaningful and appropriate conclusions. There are numerous practical benefits gained by capturing evidence. One simple but essential benefit is the ability to query a database for an annotation (an assertion about a gene product) based on a particular type of evidence. In addition, an effective evidence capture system can be used as a mechanism for filtering search results, for establishing computable rules about what data types should be associated with what types of evidence and to flag particular chains of inference that might require further review (e.g. those based purely on computational methods with no experimental verification).

A central goal of biological curation systems is to associate biological entities with descriptive information. This is achieved through curating annotations. Optimally, the associated information, i.e. the annotation, is represented with terms from a descriptive vocabulary. To document the evidence for a scientific conclusion (such as the assertion that ‘this protein has function x’), the assertion must be linked to an appropriate term that describes the evidence supporting the conclusion (such as ‘enzymatic assay’). It is possible to connect any number of evidence types with a wide range of experimentally or computationally supported assertions. However, for this to be practical, a structured, well-defined and expansive vocabulary that captures the types of evidence is needed. Ontologies are particularly well suited to this task. Ontologies allow for the detailed description of things that exist within a particular knowledge domain and provide a structural framework with relationships among descriptive terms. This enables categorization of descriptions at different levels of specificity and facilitates retrieval of data associated with ontology terms in an interpretable and computable fashion (1, 2). There are numerous examples of biological ontologies in active use. These include ontologies for phenotype (3), cell type (4), anatomy (5) and many more (6). Probably the most well-known and successful is the Gene Ontology (GO) (7) used to describe the functions, processes or cellular locations in which gene products participate or are found. Here we describe the Evidence Ontology (ECO), developed to characterize a range of evidence types in support of scientific conclusions. ECO is currently used by several biomedical resources, often in conjunction with other ontologies, to capture evidence for assertions.

## Overview of ECO

**Figure 1.**
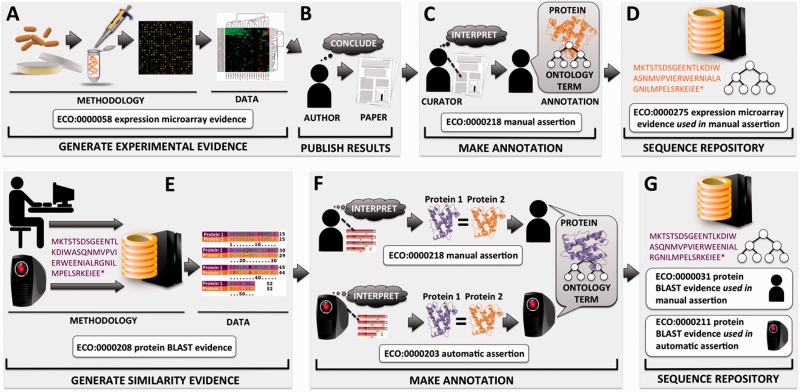
Flow of protein functional annotation and associated evidence, as represented by ECO. Intricate relationships and inputs typically represented in greater detail by other ontologies such as OBI tend to be collapsed into general summary classes in ECO. Top row (**A–D**) begins with (**A**) researcher performing a gene expression experiment and resulting data and analysis, (**B**) which are interpreted by the researcher and eventually published as conclusions. (**C**) A biological curator reads the paper and makes an association between a protein and a term from a controlled vocabulary such as the GO. (**D**) The annotation is deposited at a sequence repository along with associated evidence and assertion method. Bottom row (**E–G**) depicts two methods for asserting annotations based on similarity evidence. (**E**) Either a person or a computer compares a protein sequence with sequences in a database, which results in a hit to highly similar sequence and associated alignment data. (**F**) The machine or the human interprets the alignment, considering cutoff thresholds and other parameters. Two proteins of sufficient similarity are determined to share function, and protein 1 of unknown function will be assigned the function of protein 2 of known function. (**G**) The annotation is deposited at a sequence repository along with associated evidence and assertion method.

The ECO (http://evidenceontology.org) contains around 600 terms arranged in a hierarchy with the root node being ‘evidence’ (ECO:000000), where ‘evidence’ is defined as ‘a type of information that is used to support an assertion’. An assertion is a statement about something that is thought to be true, for example, the assignment of a function to a protein (Figure 1). The majority of evidence terms in ECO comprise either experimental or computational types, for example, ‘chromatography evidence’ (ECO:0000325) or ‘sequence similarity evidence’ (ECO:0000044), respectively. Although computational predictions and manual curation of experimental results reported in primary research literature are often the focus of discussions on annotations and their supporting evidence, ECO can indicate other types of evidence statements as well. For example, a curator may make an inference (a ‘curator inference’ evidence type, ECO:0000205) based on his or her own knowledge rather than on experimental data presented in a paper or sequence alignment. In this case, ‘inference from background scientific knowledge’ (ECO:0000001) evidence type could be cited.

In addition to describing the evidence that is used to support such assertions, ECO can be used to describe the mechanism by which an assertion is made. ECO calls this the ‘assertion method’ (ECO:0000217), defined as ‘a means by which a statement is made about an entity’. For example, whether a curator arrives at an annotation by reading about a result reported in a paper or through the process of manually evaluating pairwise sequence alignment results, ECO can capture that a manual curation method has been used (Figure 1). [For a detailed handling of how to generate curated annotations, see Balakrishnan *et al.* (8)]. Likewise, if an algorithm was used to assign a predicted function to a protein, ECO can capture that an automated computational method was used.

### Origins of ECO

The earliest versions of ECO were created from sets of evidence codes in use by the GO Consortium and its members. The GO evidence codes and the more extensive codes used by The *Arabidopsis* Information Resource (9) and FlyBase (10) provided the initial terms. The first version of ECO comprising some 127 terms was released to the SourceForge development site in early 2005 (http://purl.obolibrary.org/obo/eco/legacy).

In recent years, a growing number of ECO user requests has driven the expansion of ECO beyond GO (Table 1). Significant effort has been devoted to ECO development, resulting in the revision of most existing terms and the addition of many new terms. To further this effort, ECO development was moved from its original location as part of GO’s SourceForge tracker to a new project site (http://code.google.com/p/evidenceontology), and an independent home page was created (http://evidenceontology.org). At the time of writing, there were ∼570 terms in ECO, and many organizations are now using ECO in their evidence capture systems (Table 1).

**Table 1.** Groups and resources using the ECO or with plans to implement ECO

Group/resource	URL
AgBase	http://www.agbase.msstate.edu
AmiGO 2	http://amigo2.berkeleybop.org
The *Arabidopsis* Information Resource (TAIR)	http://www.arabidopsis.org
Ascidian Network for *in situ* Expression and Embryological Data (ANISEED)	http://www.aniseed.cnrs.fr
Bgee—a dataBase for Gene Expression Evolution	https://sourceforge.net/projects/bgee
BioModels Database	http://www.ebi.ac.uk/compneur-srv/biomodels-main
BioSapiens Network (legacy project)	http://www.biosapiens.info
European Bioinformatics Institute (EMBL-EBI)	http://www.ebi.ac.uk
The Gene Ontology (GO)	http://www.geneontology.org
IntAct Complex Portal	http://wwwdev.ebi.ac.uk/intact/complex
ISA Software Suite	http://www.isa-tools.org
Mouse Genome Informatics (MGI)	http://www.informatics.jax.org
Neural ElectroMagnetic Ontologies (NEMO)	nemo.nic.uoregon.edu
The Ontology for Biomedical Investigations (OBI)	http://obi-ontology.org/page/Main_Page
The Ontology of Microbial Phenotypes (OMP)	http://microbialphenotypes.org
Phylogenetic Annotation and INference Tool (PAINT)	http://gocwiki.geneontology.org/index.php/PAINT
PhenoScape	http://phenoscape.org
PomBase	http://www.pombase.org
Protein Information Resource (PIR)	http://pir.georgetown.edu
RNAcentral	http://rnacentral.org
*Saccharomyces* Genome Database (SGD)	http://www.yeastgenome.org/
Structure integration with function, taxonomy and sequence (SIFTS) (uses GO codes)	http://www.ebi.ac.uk/pdbe/docs/sifts/
Swiss Institute of Bioinformatics (SIB)	http://www.isb-sib.ch
The Universal Protein Resource (UniProt)	http://www.uniprot.org
UniProt-Gene Ontology Annotation (UniProt-GOA) project	http://www.ebi.ac.uk/GOA
Variation Ontology Annotation Tool VariOtator	http://www.variationontology.org/VariOtator.php
ZOOMA	http://www.ebi.ac.uk/fgpt/zooma

Visualization tool	URL
BioPortal	http://bioportal.bioontology.org/ontologies/ECO
Ontology Lookup Service (OLS)	http://www.ebi.ac.uk/ontology-lookup/browse.do?ontName=ECO
OLSVis	http://ols.wordvis.com/q=ECO:0000000
OntoBee	http://www.ontobee.org/browser/index.php?o=ECO

The table includes users who have contacted ECO with a specific development request, users of GO evidence codes transitioning to using ECO and other resources collaborating with or using ECO. While most of the users depicted use ECO to support structured queries (i.e. to group annotations), some unique examples are discussed in the text. For convenience, the ECO instances for four multi-ontology visualization tools are also listed.

### Development of ECO

ECO is developed in Open Biological Ontologies (OBO) format syntax, which is a restricted subset of Web Ontology Language (OWL; http://oboformat.org), using the ontology editor OBO-Edit (11). Term requests are received on the term tracker (https://code.google.com/p/evidenceontology/issues/list), researched and entered into the ontology as appropriate. Reasoning is performed to check term relationships and intra-ontology cross-product term validity. Releases are generated by the OBO Ontology Release Tool (http://code.google.com/p/owltools/wiki/Oor tIntro), and both OBO and OWL versions of the ontology are provided. The HermiT reasoner is run as part of the release cycle, to verify that the ontology is formally coherent, and to build the hierarchy. A continuous integration server is used to perform build and integration tests (http://build.berkeleybop.org/job/build-eco).

### Issues affecting ECO and general improvements

A number of criticisms of ECO (revision 19) were noted at an OBO Foundry (6) workshop held in 2009 (http://www.obofoundry.org/wiki/index.php/OBO_Foundry_Wo r k shop_2009) including (i) inconsistent mixing of ‘is_a’ relations based on experimental methodology, commercial platform, types of reagents used, measurement objective and analyte being measured; (ii) incomplete or entirely lacking term definitions; (iii) a lack of active development and Web presence; (iv) and a lack of interoperability, and possible overlap, with the Ontology for Biomedical Investigations (OBI) (12) and the Information Artifact Ontology (IAO) (https://code.google.com/p/information-artifact-ontology).

**Figure 2.**
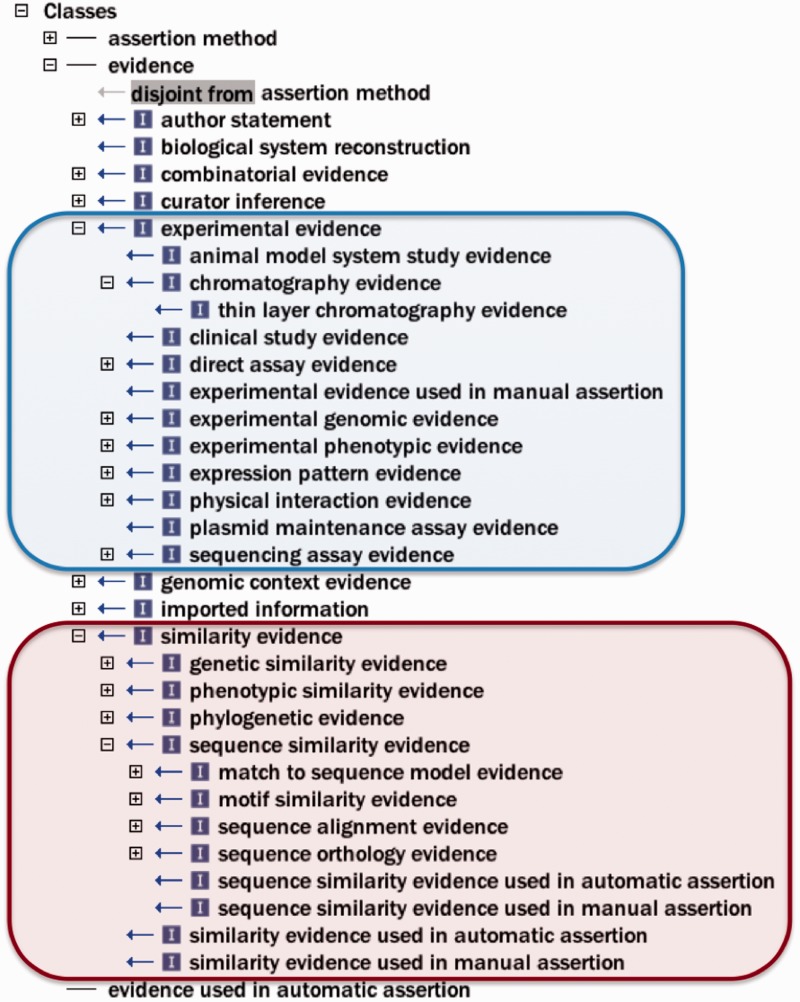
Selected ECO terms representing two major categories of evidence. The ECO class ‘experimental evidence’ (ECO:0000006) and its subclasses are circled with blue. The ECO class ‘similarity evidence’ (ECO:0000041) and some representative subclasses are circled with red.

To address these issues, and with the goal of adhering to general OBO Foundry principles for active development and interoperability (6), first general improvements (e.g. renaming terms, correcting misspelled words and adding and modifying definitions) were made to the ontology, as well as substantive efforts toward normalization (13). Improvement of term definitions was important, as the definition is the element of an ontology term that actually imparts the meaning of the term, while the term name is merely a convenient shorthand label. The root class ‘evidence’ had been previously undefined, and was given the definition ‘a type of information that is used to support an assertion’, and other definitions were written with this in mind. Figure 2 depicts some ECO classes to give the reader a sense of the types of evidence described by ECO.

### Definitions and comments

A primary target for ECO improvement was to make definitions more succinct to enhance clarity and interoperability with external ontologies, as well as to facilitate development and maintenance. To this end, definitions were edited to remove explicit references to the GO, and lengthy definitions that contained extraneous information such as usage notes and examples were shortened and formatted to conform to genus-differentia syntax. This was of particular importance because GO Consortium evidence codes are actually not directly equivalent to ECO evidence classes, but rather are analogous to ECO intra-ontology ‘evidence x assertion method’ cross products (described later). Because some long-standing ECO users include major model organism databases that perform GO annotation, as well as the GO itself, much of the GO-specific text that was removed from term definitions was retained, but appropriately rephrased, in comment fields. Such improvements allow for a term to be used in any annotation system, including one not concerned with GO evidence capture, while offering salient information to a user who is doing GO evidence capture.

### Clarifying the scope and axes of ECO

**Figure 3.**
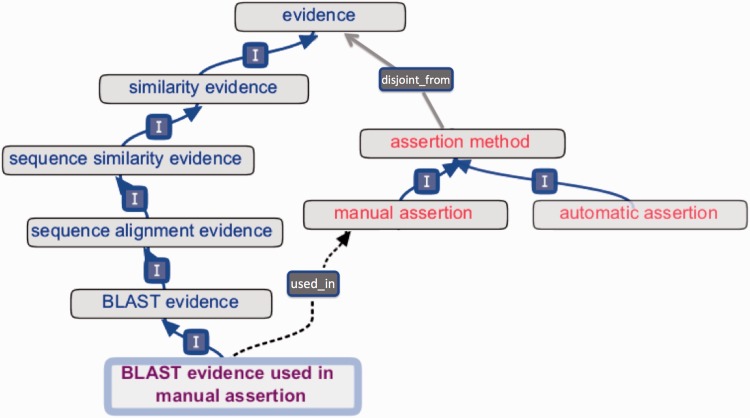
‘Evidence’ and ‘assertion method’ root classes and an internal cross product term. The evidence hierarchy (blue text) is *disjoint_from* (grey arrow) the assertion method hierarchy (red text). The cross product term ‘BLAST evidence used in manual assertion’ (purple text) descends from the evidence hierarchy (‘I’ = *is_a*), and the term’s logical definition allows a reasoner to infer the *used_in* relationship (dotted arrow) to ‘manual assertion’.

Early versions of ECO comprised a mixture of terms that conflated distinct concepts, including ‘origin’ of evidence (e.g. type of assay or analysis generating data), ‘type’ of information contributing to evidence (e.g. piece of data or result of a data analysis), ‘evaluation’ or ‘assessment’ yielding evidence (e.g. data analysis culminating in evidence), ‘assignment’ of evidence in an annotation (e.g. by a human or a machine) and ‘inference’ drawn from evidence (e.g. a conclusion arrived at after weighing evidence). Such mixing of ideas was present in term names and definitions and was unclear in inheritances. To enhance conceptual clarity and usability, as well as to facilitate future development and interoperability with existing ontologies, it was essential to distinguish among the different interpretations of the meaning of evidence. Two axes were established for the organization of all ECO terms (Figure 3). The first, the ‘evidence’ axis, captures the type of evidence that is derived from an inquiry and that is used to support an assertion (Figures 1 and 3). The second axis, the ‘assertion method’, distinguishes between manual (human curated) and automatic (computer curated) methods of making assertions (Figures 1 and 3).

It is possible to create new terms from cross products of evidence types and assertion types, using the *used_in* relation to connect evidence to assertion method (Figures 1 and 3). For example, to describe that an analysis using basic local alignment search tool (BLAST) (14) had been performed and that a person had reviewed the resulting evidence before annotating protein x with function y, one could use the ‘BLAST evidence used in manual assertion’ (ECO:0000030) term, which is defined as a ‘BLAST evidence’ (ECO:0000206) that is *used_in* ‘manual assertion’ (ECO:0000218) (Figure 3). This textual definition is accompanied by a computable logical definition expressed as an OWL equivalence axiom:

ECO_00000030 EquivalentTo ECO_0000206 and *used_in* some ECO_0000218

Such compositional terms are all descended from the ‘evidence’ root class (e.g. ‘BLAST evidence’) and connected with the ‘is_a’ relationship. Although asserted as members of the ‘evidence’ hierarchy, they are differentiated by being *used_in* either manual assertion (ECO:0000218) or automatic assertion (ECO:0000203). Currently, the *used_in* relation is defined as ‘a relation connecting a piece of evidence to an assertion method, where that assertion method is supported by the evidence’ and is restricted to hold between these two ECO classes. In the future, a more general-purpose relation may be selected from an ontology such as the Relationship Ontology (15), IAO or OBI.

### External ontology cross references

GO evidence codes have been used extensively to divide most annotations into two broad categories: those based on experimental evidence, and those based on computed or automatic evidence, the latter of which is often associated with high-throughput data. Automatically curated annotations can be identified in the GO annotation set primarily by the ‘IEA’ (inferred from electronic annotation) abbreviation. Such an evidence code is referenced by ECO through the common practice of assigning a database cross-reference (‘dbxref’), where a term in one ontology is linked to an analogous term in another ontology or other resource. In this particular case, the ECO term ECO:0000501 ‘evidence used in automatic assertion’ is the cross product of ‘evidence’ (ECO:0000000) and *used_in* ‘automatic assertion’ (ECO:0000203) and carries the dbxref ‘GOECO: IEA’. In another example dealing with experimental evidence, ECO:0000353 ‘physical interaction evidence used in manual assertion’ is analogous to the GO code ‘IPI’ (inferred from physical interaction) and so contains the cross-reference ‘GOECO:IPI’.

Beyond encoding such one-to-one dbxrefs, external resources such as the GO can map a combination of terms to an equivalent ECO class. The GO Consortium uses ‘GO references’ (GO_REF; http://www.geneontology.org/doc/GO.references) to describe various curation methodologies that can be captured in the ‘Reference’ field of a GO annotation. A specific combination of a GO_REF and a GO evidence code is considered equivalent to a particular granular ECO class. For example, whereas the GO abbreviation ‘IEA’ by default maps to ECO:0000501 ‘evidence used in automatic assertion’, the ‘IEA’ evidence code in combination with GO_REF:0000002 [which references InterPro-created GO annotations (16)] maps to the more granular ECO class ECO:0000256 ‘match to sequence model used in automatic assertion’. The GO provides a mapping table that translates such GO code-GO_REF pairs to their equivalent ECO classes (http://purl.obolibrary.org/obo/eco/gaf-eco-mapping.txt). Other ontologies or vocabularies are free to map to ECO terms in a similar fashion.

#### Browsing ECO

ECO can be browsed on various different web-based ontology platforms, including the Ontology Lookup Service (OLS), OLSVis, BioPortal and OntoBee (Table 1). All of these sites allow browsing of ECO itself, but not associated data.

The GO Browser AmiGO 2 allows for faceted browsing in its search interface (17)—the full ECO hierarchy is used. Thus, for example, if a user selects ‘experimental evidence’ in the evidence facet, the annotations selected are both those directly annotated to this type (abbreviation ‘EXP’/ECO:0000006) as well as annotations that use any descendant of this type.

## Applications of ECO

Many of the groups known to use ECO are depicted in Table 1. Below are descriptions of how some representative groups are using ECO.

### UniProt

The original UniProtKB evidence types have been replaced with terms from ECO. These are already available in the UniProtKB XML and will shortly be visible in the flat file format as well. Novel ways of mapping and extending ontologies have been discussed with ECO and the GO Consortium to ensure appropriate development for UniProtKB annotation.

The UniProt-Gene Ontology Annotation (UniProt-GOA) project provides >169 million manual and electronic evidence-based associations between GO terms and 26.5 million UniProtKB proteins covering >411 000 taxa (18). Of these, manual annotation provides 1.4 million annotations to ∼260 000 proteins. Since 2010, UniProt-GOA has supplied GO annotations in a Gene Product Association Data (GPAD) file format, which allows inclusion of ECO terms. As previously mentioned, selected ECO terms are cross referenced to their corresponding GO codes, so even if evidence for annotations was supplied to UniProt as GO codes, the GPAD file will display the appropriate equivalent ECO term. Thus, UniProt annotations can be grouped by leveraging the structure of ECO. UniProt will shortly transition to curating GO annotations using ECO codes in preference to GO evidences.

### The GO

The GO project (7) captures evidence information not only to produce comprehensive high-quality annotation but also for quality control. For example, the GO curatorial processes use evidence to support computable rules about the kinds of information that are required to be associated with different types of evidence. One such rule states that annotation of a protein that is based on an alignment with another protein requires that the identity of the matching protein be captured. Use of ECO terms can effectively allow automated quality control checks to insure that all required information is supplied for a given type of annotation. In addition, evidence types are also used by GO as a quality control check for annotation consistency. For example, the GO restricts usage of evidence based on expression pattern to annotations with terms from the biological process ontology. Thus, annotations to terms from either of the other two GO ontologies (molecular function and cellular component) would be flagged as suspect. In a final example, the GO uses evidence tracking to prevent circular annotations grounded only in computational predictions. Evidence tracking allows chains of evidence to be computationally examined to insure that experimental evidence forms the basis for inferential annotations. For example, annotations made with the ISA GO evidence code (‘inferred from sequence alignment’) require the inclusion of a stable database identifier that identifies the similar gene/gene product. To avoid circular inferences, the similar gene must be experimentally characterized.

The GO has also developed a set of computable rules [see (7)] for determining whether a particular usage of an ECO evidence type is valid and is accompanied by sufficient information. These are available from http://www.geneontology.org/GO.annotation_qc.shtml. At present, there are 11 rules implemented, three rules approved and five rules proposed. For example, rule GO_AR:0000018 states that every physical interaction evidence (GO IPI/ECO:0000021) is accompanied by a supporting interacting protein or other molecule.

To amplify the benefits of experimental knowledge that curators capture, the GO Consortium is using a phylogenetic tree-based approach to generate manually reviewed, homology-based annotations for a broad range of species (19). It is based on an explicit evolutionary model and uses an intuitive graphical output facilitating the rapid identification of homology sets by curators. This phylogenetic annotation methodology necessitated a new set of evidence terms to capture the inference process. These phylogenetic evidence types are biological aspect of ancestor (GO IBA or ECO:0000318 ‘biological aspect of ancestor evidence used in manual assertion’), where the biological characteristics in descendent sequences are inferred from the biological characteristics of the ancestral sequence, and conversely, biological aspect of descendant (GO IBD or ECO:0000319 ‘biological aspect of descendant evidence used in manual assertion’), whereby the characteristics of an ancestral sequence are inferred through the experimental characterization of an extant descendant sequence. To cover loss of function, there are also inferences made by determination that key residues are missing (GO IKR or ECO:0000320 ‘phylogenetic determination of loss of key residues evidence used in manual assertion’), and divergence (GO IRD or ECO:0000321 ‘rapid divergence from ancestral sequence evidence used in manual assertion’) by inference from long phylogenetic tree branch lengths following a duplication event. For an example of how prevalent the use of these types of evidence is, in September 2013, the GO database contained 68 326 annotations with the IBA evidence code covering 82 species. In March 2014, that number had risen to 132 841 annotations and continues to increase rapidly.

### The Ontology of Microbial Phenotypes

Characterization of phenotypes is important for many biomedical research applications including clinical identification of microbes, biotechnological applications and for determining protein function through genetic manipulation. The Ontology of Microbial Phenotypes (OMP; http://microbialphenotypes.org, manuscript in preparation) has been developed to standardize phenotypic information capture for diverse microbes. There are numerous classes of evidence associated with the study of microbes including evidence resulting from growth assays, motility assays, biochemical tests and antibiotic resistance tests. Just as it is now routine to make an assertion that a particular gene product has a particular function, one can also make an assertion that a particular genotype displays a particular phenotype. The phenotype can be captured using an OMP term, and the evidence for determining the phenotype will be captured with an ECO term. For example, if a mutation causes normally motile cells to have a decreased ability to swim, one will capture this by linking the OMP term ‘decreased motility’ to an identifier for the mutant strain. The evidence for this annotation would be captured with an ECO term such as ‘motility stab test evidence’. Thus, researchers will be able to track which types of evidence were used to assess which phenotypes. It is important to note that phenotypes can also be predicted from whole-genome or transcriptome analysis of individual strains, and ECO is well suited to track these computational evidence types, just as it does for automated annotation of gene function. The application of ECO to capturing evidence associated with phenotypic study, and the resulting annotation of mutant phenotypes with these ECO terms, will facilitate studies in a broad area of applied research. An implementation of ECO can be browsed at the OMP wiki (http://microbialphenotypes.org/wiki/index.php/Cat eg o r y : ECO:0000000_!_evidence).

## Future work

### Ongoing term development and ontology revision

Like all ontologies, there remain areas in ECO that are incomplete and under development. Work continues on normalization, adding new terms as requested by users, and collaborating with interested databases and resources to achieve common standards.

### Documentation for users

The ECO contains a rich and growing number of terms to annotate myriad types of evidence. Because definitions are by design succinct, and because experimental and computational methods are both complex and nuanced, users will benefit from enhanced documentation that includes term usage explanations and examples. Work is underway to more extensively document all aspects of ECO term creation and usage.

### Complex term creation

There is an increasing need, as reflected by user requests, for more granular ontology terms. A decade ago, ECO was adequately suited to capture general high-level classes of evidence. Now, however, there is a growing need for highly specific terms, and sometimes combinations of terms that might be viewed as rather complex workflows. For example, recently a user requested the following term: ‘sub-cellular fractionation and subsequent protein identification using 2-D liquid chromatography MS/MS, Orbitrap electrospray ionization followed by high-throughput analysis of the results to determine sub-cellular localization (GO Cellular Component)’. This evidence type encapsulates a chain of events, which is difficult and sometimes impossible to fully capture as an equivalence axiom using OBO format. To better model the experimental complexity, future ECO development will be migrated to OWL, which allows for greater expressivity. This will not affect the majority of ECO users, who will still have the option of either OBO or OWL versions.

One advantage of the increased expressivity of OWL is the ability to directly model chains of evidence. Patterns involving sequences of arbitrary length can be easily modeled in OWL using nested class expressions—for example, an evidence chain involving sequence similarity based on mutant phenotype can be described with an expression ‘based on’ some (‘sequence similarity’ and ‘based on’ some ‘mutant phenotype’). Even if the need for this complex modeling is not immediately required, migrating the base version of the ontology to OWL will allow the use of tooling such as OWL reasoners to help maintain the ontology.

### Collaboration with OBI

Another promising area of development that will enable ECO to include complex terms lies in recent collaborative work undertaken with developers of the OBI (12). OBI is similar in content, and in some cases parallels ECO, in that it includes a classification of different types of instrument, protocols, analyses, materials and so on. However, it is formally orthogonal because ECO deals with evidence types, which are disjoint from the actual experiments performed. ECO classes are information artifacts, and most ECO evidence types can be defined as specified outputs of OBI ‘planned process’ and its subtypes, including ‘assay’ and ‘data transformation’. Since 2011, ECO has been working with OBI to leverage these attributes through two means. First, ECO can leverage existing instruments and methods described by OBI to facilitate rapid term development. For example, the ECO:0000224 term ‘SOLiD sequencing evidence’ can be created as an output of the OBI ‘planned process’ subclass ‘SOLiD sequencing’. This will allow an efficient division of responsibilities and avoid duplication of effort. Second, multiple instruments and methods, whose use culminate in complex evidence types, could be modeled in OBI as multiple ontology terms with numerous relationships, along with a simpler summary-type class. This OBI term could be imported by ECO, i.e. connected to a new ECO term via a bona fide relationship. Established protein and model organism resources are the ultimate consumers of ECO, and they require straightforward evidence classes that are easy to incorporate within their existing frameworks, so this approach offers promise as a solution.

At present, ECO contains some evidence terms that arise from processes not yet instantiated in OBI. Conversely, OBI has numerous classes whose outputs are not represented in ECO. Where possible, ECO makes use of the OBI ID as definition provenance (for example, in the definition of ‘ECO:0000224’ SOLiD sequencing evidence). We acknowledge that this is a weak form of integration, and we aim to continue to work with OBI to increase interoperability and synergistic growth where possible.

### Collaboration with IAO

The IAO is an offshoot of OBI designed to model pieces of information, including those pertaining to biomedical investigations. It may be the case that ECO will be conceived of as an extension to IAO, with the root node of ‘evidence’ being a subtype of information artifact.

### Use of an upper ontology

Currently, ECO is neutral with respect to upper ontologies, and could potentially be used in combination with different upper ontologies such as Basic Formal Ontology (BFO) (20) or the Semanticscience Integrated Ontology (21). If ECO formally becomes an extension of IAO, then it will by inheritance also be an extension of BFO. However, we do not anticipate any major modeling changes required for conformance, and in principle it would still be possible to extract an upper-ontology neutral subset of ECO for use in other frameworks.

### Evidence quality and ECO

With respect to weighting types of evidence, clearly there is a perceived difference in quality between different types of evidence. For example, experimental assays are generally viewed as the gold standard for evidence and thus more reliable than other types of evidence such as computationally derived sequence-based methods. However, in fact when examined carefully, there is huge variation in quality and reliability in all types of evidence, both experimental and computational. It is not possible, based on evidence type or assertion method alone, to determine the quality of evidence. Thus, ECO evidence terms do not carry implicit assumptions about the quality of a particular type of evidence or measures of confidence in different types of evidence. Each method has inherent strengths and challenges for identification of underlying biological knowledge at a certain level of granularity. The kind of evidence, along with the selection of the annotation terminology that is consistent with the level of granularity uncovered by the method, combines to provide a level of confidence in the assertion.

Thus, an independent and orthogonal method must be developed to capture annotation evidence quality and confidence. Although this is not currently within the scope of ECO, we are eager to work with the broader community to establish ways in which this can be captured by making use of resources such as ECO. For example, at the 2012 International Society for Biocuration meeting, ECO developers participated in a workshop hosted by researchers from the Swiss Institute of Bioinformatics titled ‘Quality information in support of annotations’ (http://wiki.isb-sib.ch/biocuration/Workshop_follow-up). It was clear that there is a desire in the community to capture quality/confidence metrics. Although the best way to achieve this has not been decided, ECO will be part of the community effort to solve this challenge.

## Conclusion

The past several years have seen ECO grow into an independent, robust and actively developed ontology. ECO has a centralized Web presence (http://evidenceontology.org) and development site (http://code.google.com/p/eviden ceontology) that is being used by the community. Initial improvements to ECO included structural modifications to the ontology, batch term renaming, spelling corrections, term merges, term obsoleting, completing definitions and creating a new root class, as well as generation of internal cross products. Work on normalization continues. These changes were essential to promoting usability of ECO and have resulted in the growth of our user community. Several major annotation resources now use ECO for facilitating information retrieval and quality control. We continue to pursue several areas of development including collaborations with orthogonal ontologies such as OBI. ECO development will continue to be responsive to user needs, and we welcome user feedback and requests at the ECO tracker site (http://code.google.com/p/evidenceontology/issues/list).
